# Improving and degrading the oxygen exchange kinetics of La_0.6_Sr_0.4_CoO_3−*δ*_ by Sr decoration†

**DOI:** 10.1039/d2ta09362f

**Published:** 2023-02-13

**Authors:** Matthäus Siebenhofer, Christoph Riedl, Andreas Nenning, Werner Artner, Christoph Rameshan, Alexander Karl Opitz, Jürgen Fleig, Markus Kubicek

**Affiliations:** a Institute of Chemical Technologies and Analytics, TU Wien Vienna Austria matthaeus.siebenhofer@tuwien.ac.at; b Centre for Electrochemistry and Surface Technology, CEST Wr. Neustadt Austria; c X-Ray Center, TU Wien Vienna Austria; d Chair of Physical Chemistry, Montanuniversität Leoben Leoben Austria

## Abstract

Minimizing the overpotential at the air electrode of solid oxide fuel cells (SOFC) is one of the key challenges regarding a broad applicability of this technology. Next to novel materials and geometry optimization, surface modification is a promising and flexible method to alter the oxygen exchange kinetics at SOFC cathode surfaces. Despite extensive research, the mechanism behind the effect of surface decorations is still under debate. Moreover, for Sr decoration, previous studies yielded conflicting results, reporting either a beneficial or a detrimental impact on the oxygen exchange kinetics. In this contribution, *in situ* impedance spectroscopy during pulsed laser deposition was used to investigate the effect of Sr containing decorations under different deposition conditions. Depending on deposition temperature and interactions with the gas phase, opposing effects of Sr decoration were found. In combination with near-ambient pressure X-ray photoelectron spectroscopy and non-ambient X-ray diffractometry, it was possible to trace this phenomenon back to different chemical environments of the surface Sr. At high temperatures, Sr is deposited as SrO, which can have a beneficial effect on the oxygen exchange kinetics. At low temperatures, SrCO_3_ adsorbates are formed from trace amounts of CO_2_ in the measurement atmosphere, causing a decrease of the oxygen exchange rate. These results are in excellent agreement with the concept of surface acidity as a descriptor for the effect of surface decorations, providing further insight into the oxygen exchange kinetics on SOFC cathode surfaces and its degradation. In addition, this study shows that Sr segregation itself initially does not lead to performance degradation but that segregated SrO readily reacts with acidic compounds, reducing the catalytic capability of mixed conducting oxides.

## Abstract 2

i-PLD measurements reveal beneficial effects of SrO at high temperatures and detrimental effects of CO_2_ adsorbates at low temperatures.

## Graphical abstract

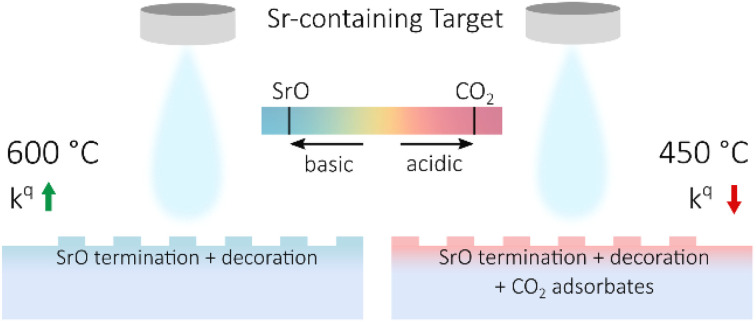

**10th anniversary statement**

Since its beginning, the Journal of Materials Chemistry A has played an essential role for researchers aiming at an advanced understanding of the oxygen exchange reaction on mixed conducting surfaces, both by harboring excellent papers from research groups all over the world and by ensuring the highest possible quality during review and publication processes. From exploring the surface chemistry of oxides and its evolution at high temperatures to investigating the oxygen exchange reaction on truly pristine oxide surfaces with *in situ* impedance spectroscopy during pulsed laser deposition, the Journal of Materials Chemistry A has published many essential findings, also of our research group. Hence, we are delighted to continue this cooperation and to contribute an article to this anniversary issue. We are excited to see in which direction research will lead the development of novel energy materials and are eagerly looking forward to expanding our horizons when reading the Journal of Materials Chemistry A.

## Introduction

The kinetics of the oxygen exchange reaction (OER) on surfaces of solid oxide fuel cell (SOFC) cathodes and its degradation have posed a sizeable challenge for researchers attempting to lower the operating temperature of SOFCs and to optimize their efficiency.^1–5^ While La_0.6_Sr_0.4_CoO_3−*δ*_ (LSC) is a promising SOFC cathode material due to its high conductivity and fast oxygen exchange kinetics,^6–9^ its tendency towards performance degradation under operating conditions limits its applicability.^2,10^ As main reasons for the observed degradation, two phenomena are frequently mentioned in literature. On the one hand, detrimental impurities in the gas phase such as S, CO_2_, Cr or Si^2,11–13^ have been reported to cause a substantial decrease of the OER rate of LSC or similar mixed conducting oxides. On the other hand, Sr segregation has been suggested to inhibit the oxygen exchange kinetics of LSC, *e.g.* by the formation of secondary phases.^14–17^

However, different and partly contradicting results can be found in literature, particularly on the effect of Sr at the surface. It has been recently established that the equilibrium surface of LSC under SOFC operating conditions is mainly SrO terminated and that there is a general tendency of the Sr dopant to segregate to the surface.^18–22^ The effect of this SrO termination layer itself on the oxygen exchange kinetics is not fully clear. Rupp *et al.* performed several experiments (*e.g. in situ* decoration with Sr at 450 °C and 0.5 mbar *p*(O_2_) or annealing of LSC samples after removal of the SrO termination layer by water) and concluded that an SrO termination layer has a detrimental effect on the OER kinetics. During the discussion of underlying mechanisms, the focus was placed mainly on the coverage of active sites. Similar results were also reported for Sr decoration on La_0.8_Sr_0.2_FeO_3−*δ*_ with atomic layer deposition.^23^ These results are in line with the widely accepted model that Sr segregation and Sr enrichment on perovskite surfaces is one of the main reasons for the performance degradation under operating conditions.

In contrast to these results, Mutoro *et al.* decorated LSC with larger amounts of Sr and observed an increase of the surface exchange coefficient, indicating that surface Sr can also accelerate the oxygen exchange kinetics. They suggested an active phase formation, possibly a Ruddlesden Popper phase between LSC and Sr particles.^24,25^ In light of recent results on surface decoration experiments on Pr_0.1_Ce_0.9_O_2−*δ*_ (PCO), where the acidity of decorated oxides was established as a descriptor of their effect on the oxygen exchange kinetics of PCO,^26,27^ these conflicting reports call for a reinvestigation of SrO decoration on LSC and a reinterpretation of results regarding degradation due to Sr segregation.

In this contribution, impedance spectroscopy during pulsed laser deposition (i-PLD) is employed to examine the effect of Sr containing decorations on dense LSC thin films. Interestingly, the experimental results confirm both previously described trends, although at different temperatures. At high temperatures, SrO is the dominating surface oxide, which improves the oxygen exchange kinetics of LSC. At low temperatures, the majority of the decorated Sr is bound in the form of surface carbonates, which causes a strong decrease of the oxygen exchange kinetics. A detrimental effect of such surface carbonates was also observed for similar SOFC cathode materials^13,28–32^ and the suggested underlying processes primarily involve competitive adsorption of CO_2_ and O_2_ ^29^ and blocking of surface oxygen vacancies.^13^

By the combination of our i-PLD results and analytical techniques (near-ambient pressure XPS and non-ambient X-ray diffractometry), this study not only identifies possible reasons behind different results from earlier studies about the effects of surface Sr,^23,25,33^ but also brings these results in the context of surface acidity,^26,27^ thereby further advancing the understanding of the oxygen exchange reaction and of degradation processes on LSC, in particular with regard to the true effects of Sr segregation.

## Experimental

### Preparation of half-cell substrates

Before performing i-PLD measurements, current collector grids and counter electrodes were deposited on (001) oriented yttria stabilized zirconia (YSZ, 5 × 5 × 0.5 mm^3^, 9.5 mol% Y_2_O_3_, Crystec GmbH, Germany) single crystals. Ti/Pt grids (5/300 nm) were prepared on both sides by lift-off photolithography and metal sputtering (BalTec MED 020, Leica Microsystems GmbH, Germany). During i-PLD measurements, this grid ensures that the whole sample surface is homogeneously polarized and that only the area in direct contact to the YSZ electrolyte is electrochemically active. The exact grid size and the grid-free area were determined by measurements with an optical microscope.

On one side of the YSZ single crystals, a nano-porous LSC counter electrode with very low polarization resistance was deposited^6,34^ by PLD (200 nm thickness, 9000 pulses, 450 °C, 0.4 mbar, 5 Hz, 1.1 J cm^−2^, 5.0 cm target to substrate distance). All PLD depositions were performed with a KrF excimer laser (Lambda Physics, COMPex Pro 201, *λ* = 248 nm). On the other sample side, the thin film working electrodes were deposited during i-PLD. Directly prior to i-PLD measurements, the sample edges were carefully ground to avoid short circuiting from the growing working electrode to the counter electrode *via* Pt or LSC remnants.

### *In situ* impedance spectroscopy during pulsed laser deposition

**Fig. 1:**
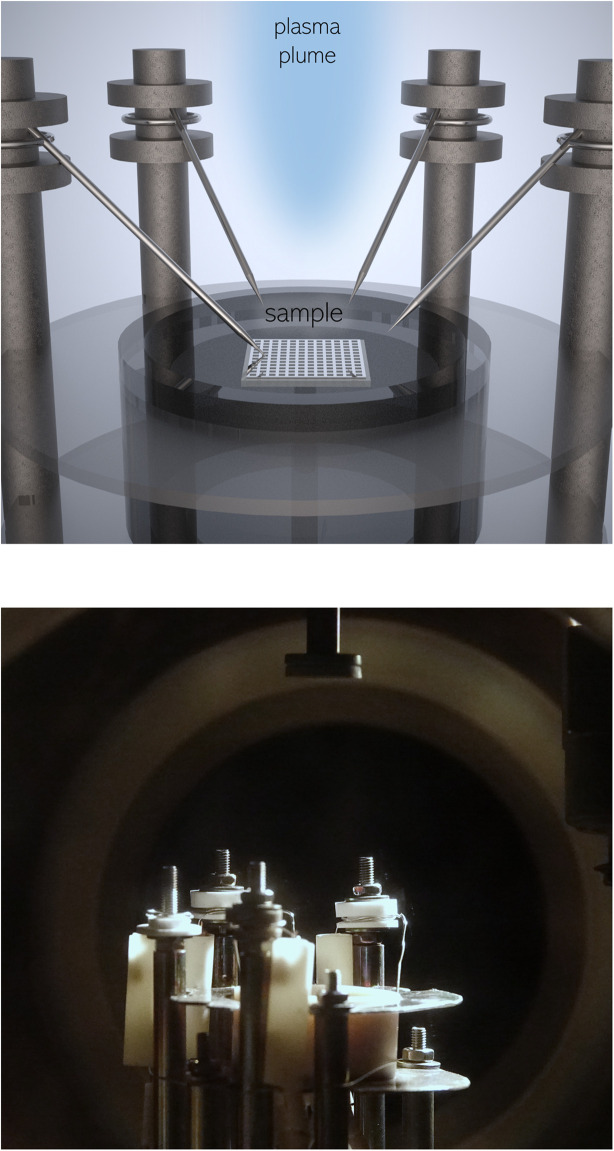
Close-up visualization of the i-PLD heating stage as well as a photograph inside of the PLD chamber with a mounted target.

This technique and the corresponding setup were previously described in detail.^7,34,35^ A customized heating stage for the PLD chamber (Huber Scientific, Austria) was used for all i-PLD experiments in this study (see Fig. 1). The sample stage consists of an Al_2_O_3_ block with embedded Pt wires for resistive heating. The top is brushed with Pt paste and the sample is placed on the heater, which automatically guarantees electrical contact to the counter electrode. The current collector on top of the sample is contacted with a Pt/Ir needle, which remains on the sample during the whole i-PLD experiment. The LSC target was prepared by a modified Pecchini route^7^ and the target stoichiometry was adapted until the thin film stoichiometry (determined by LA-ICP-MS) was satisfying.^7^ Prior to LSC deposition, the target was ground and preablated for 1 min at 5 Hz and for 1 min at 2 Hz. The PLD chamber was evacuated to a base pressure below 10^−4^ mbar and the oxygen partial pressure needed during i-PLD experiments was adjusted with O_2_ gas flow (all gases used in the experiments were 5.0 purity measurement gases, ≤0.4 ppmv CO_2_, Messer GmbH, Austria). The sample temperature was controlled *via* the high frequency intercept of the recorded impedance spectra, which corresponds to the sum of the setup wiring resistance (measured beforehand), the ionic conduction resistance of the electrolyte (strongly temperature dependent^36^) and the resistance of the Ti/Pt grid (estimated based on the grid geometry^34^). Due to the Arrhenius-type activation of the ionic conductivity of YSZ, this method allows a precise measurement and regulation of the sample temperature.

At the beginning of all experiments, the deposition rate was measured with a quartz balance inside of the PLD chamber to account for slight variations of the laser spot size. Afterwards, a dense LSC thin film was grown until the surface exchange resistance remained constant (indicating that the surface area of all film parts directly in contact with the electrolyte was electrochemically active and that no in-plane conduction contributions affected the measurement). The LSC working electrode deposition was performed at 600 °C, 0.04 mbar, at a laser frequency of 2 Hz, a laser fluence of 1.1 J cm^−2^ and a target to substrate distance of 6.0 cm. The sample impedance was tracked with an Alpha-A High Performance Frequency Analyzer and Electrochemical Test Station POT/GAL 30V/2A setup (Novocontrol Technologies) in a frequency range from 10^6^ to 10^−1^ Hz, an AC RMS voltage of 10 mV and a resolution of 5 points per frequency decade. The growth process of LSC itself has been investigated previously^7,34^ and is not subject of this study.

Decorations were then performed by deposition from a Sr-containing target. The target was manufactured by calcination (12 h, 1000 °C under oxygen flow) and subsequent cold isostatic pressing and sintering (12 h, 1200 °C under oxygen flow) of SrO powder (Sigma Aldrich, 99.5%). The target surface was carefully ground before every experiment and XRD measurements on a ground surface revealed a target composition of 40% SrO and 60% Sr(OH)_2_. Again, the deposition rate was determined with a quartz balance before each experiment and recalculated to monolayers of SrO, with two monolayers corresponding to a layer thickness of one unit cell. During standard decoration, single pulses were deposited onto the LSC surface and impedance spectra were measured after each pulse. For saturation experiments, also higher pulse numbers and Sr amounts were deposited. For decorations in CO_2_ atmosphere, CO_2_ gas was fed into the PLD chamber and the pressure change was recorded to determine the exact amount of CO_2_ in the PLD chamber (in the range of 0.0015 to 0.0260 mbar for a background pressure of 0.04 mbar O_2_).

### Near-ambient pressure X-ray photoelectron spectroscopy (NAP-XPS)

X-ray photoelectron spectroscopy was performed in a lab-based NAP-XPS setup with a PHOIBOS NAP photoelectron analyzer (SPECS, Germany) and a monochromated Al K-α XR 50 MF microfocus X-ray source. The sample was mounted on a customized sample holder with a 4.5 × 4.5 mm^2^ hole for laser heating with a near-infrared diode laser.^37^ Electrical contact was established with Pt/Ir wires holding the sample in the sample stage. Again, the temperature during XPS measurements was controlled *via* the real-axis intercept of the impedance curve (see above). XPS spectra were recorded at different oxygen partial pressures, pressure gauges were used to determine the current pressure. Similarly to i-PLD measurements, a controlled amount of CO_2_ was fed into the XPS chamber to observe its effect on the surface chemistry. XPS spectra were collected at an analyzer pass energy of 30 eV, which provided a reasonable balance of count rate and energy resolution.

## Results

### *In situ* Sr decoration in different conditions

**Fig. 2:**
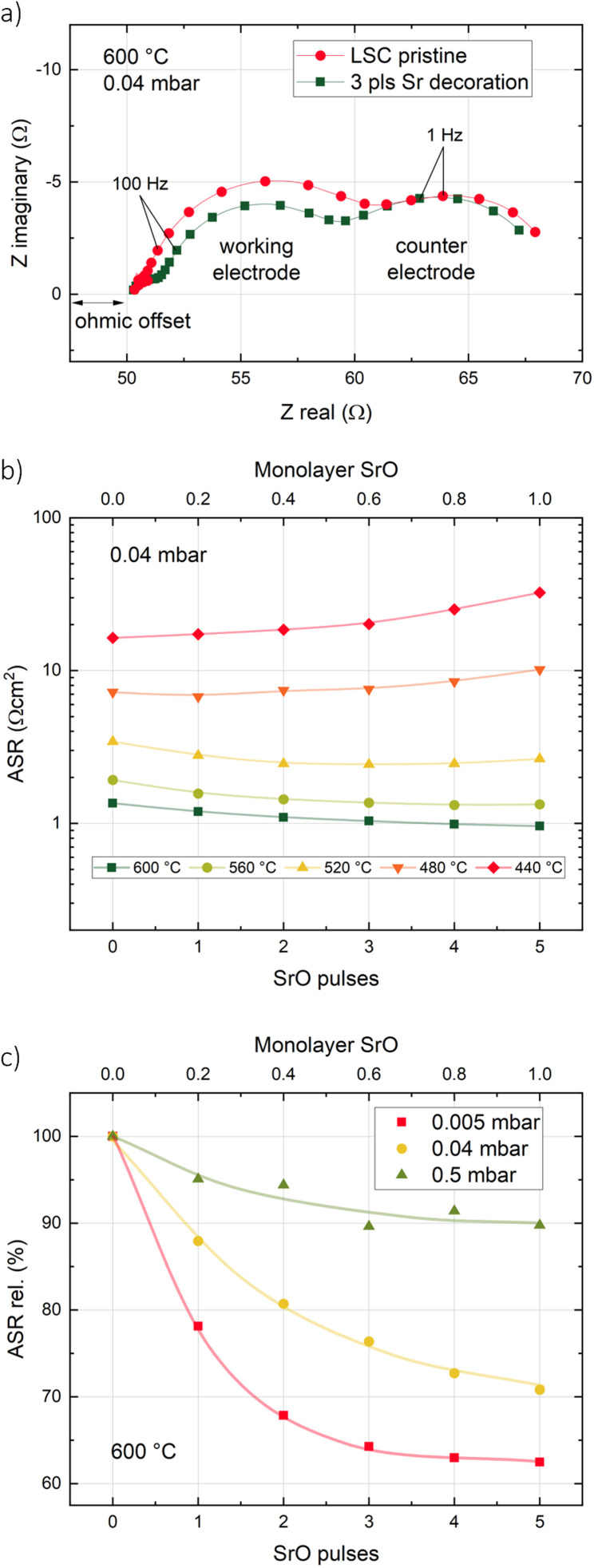
(a) Exemplary impedance curves of pristine and Sr decorated LSC at 600 °C and 0.04 mbar. (b) Evolution of the area specific surface exchange resistance of a dense LSC thin film at different temperatures and 0.04 mbar *p*(O_2_) upon *in situ* decoration from a Sr-containing target. (c) Evolution of the relative area specific surface exchange resistance at different partial pressures. Values are normalized to the initial resistance without Sr decoration. Lines are a guide to the eye.

Pristine LSC thin films were decorated with different amounts of SrO (corresponding to up to 1 monolayer) at different temperatures and oxygen partial pressures. AFM images of the surface morphology of LSC thin films before and after decoration show a granular surface with no significant changes induced by the addition of 1 monolayer of SrO (see ESI 1†). Exemplary impedance curves as well as temperature and *p*(O_2_) dependencies are shown in Fig. 2. A detailed interpretation of the corresponding impedance spectra can be found elsewhere.^34^ Essentially, a medium frequency arc can be attributed to the oxygen exchange resistance at the surface and its value is referred to the active area (LSC directly on YSZ). From the measurement results, it is clearly visible that a nominally identical decoration from the same target can have opposite effects depending on the decoration temperature. At high decoration and measurement temperatures (600 °C), Sr decoration improves the oxygen exchange kinetics, *i.e.* it decreases the surface exchange resistance, while the inverse effect occurs at low temperatures (440 °C). Moreover, the oxygen partial pressure during decoration at 600 °C has a substantial effect on the amount of improvement of the oxygen exchange kinetics. In general, the lower the deposition (and measurement) oxygen partial pressure, the stronger is the improvement.

### Temperature dependency

**Fig. 3:**
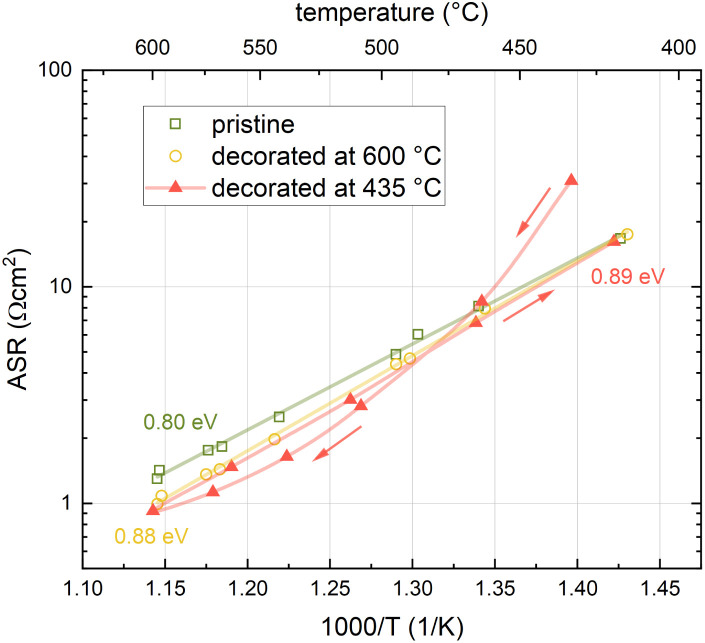
Arrhenius-type diagram of the area specific surface exchange resistance of a dense LSC thin film decorated with 1 monolayer of SrO once at high (600 °C) and once at low temperature (435 °C) at 0.04 mbar *p*(O_2_).

For an in-depth investigation of this phenomenon, a detailed activation energy analysis was performed for LSC decorated with 1 monolayer of SrO at high temperatures and LSC decorated with 1 monolayer of SrO at low temperatures (Fig. 3). These measurements were performed on one and the same sample in 0.04 mbar oxygen. First, LSC was grown and the activation energy of its surface exchange resistance was measured. Then, Sr was decorated at 600 °C and the activation energy analysis was repeated. Afterwards, a pristine LSC surface was restored by the deposition of 10 nm LSC at 600 °C. Subsequently, SrO was decorated at 435 °C and again, the activation energy was measured. The measurements yielded a very low activation energy of 0.80 ± 0.02 eV for pristine LSC. This is in agreement with previous i-PLD studies, showing that the activation energy of the surface exchange resistance continuously decreases with decreasing oxygen partial pressure.^7,38^ Upon decoration at 600 °C, the surface exchange resistance dropped, in agreement with Fig. 2, and in addition, the activation energy increased slightly to 0.88 ± 0.02 eV. This change of the activation energy is surprising and will be discussed in more detail below. As the activation energies were measured from high temperature to low temperature and back, we can rule out any irreversible, temperature dependent changes of the surface chemistry of high T decorated LSC.

For low T decorated LSC, the temperature dependence is completely different. When LSC was decorated at 435 °C, the surface exchange resistance increased as expected, however, when increasing the temperature, the resistance decrease was much stronger than anticipated. When reaching 600 °C, the resistance was virtually identical for both decoration temperatures. This also holds for the activation energy when cooling from 600 °C, indicating that low T decorated LSC undergoes a transition at higher temperatures and finally reaches the same chemical state as high T decorated LSC.

### Surface chemistry of decorated LSC thin films

**Fig. 4:**
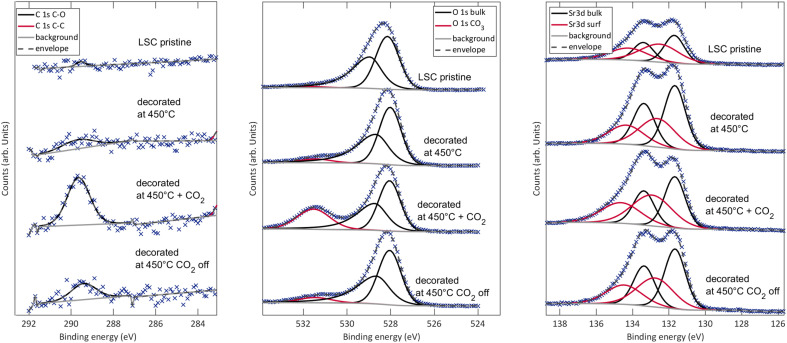
XPS results for the C, O and Sr signals on pristine LSC surfaces and on low T decorated surfaces before, during and after CO_2_ addition in the XPS chamber.

This phenomenological observation already suggests that the effect of surface Sr on the oxygen exchange kinetics of a pristine LSC surface strongly depends on the chemical state of the Sr. For an in-depth analysis of the surface chemistry of Sr-decorated LSC, XPS measurements were performed. The results show an unusually high amount of carbon on the low T decorated sample compared to the pristine sample and a concomitant additional oxygen species corresponding to CO_3_^2−^. To confirm a correlation with carbonate adsorbates, CO_2_ was introduced deliberately into the XPS chamber. The results of the according XPS measurements are shown in Fig. 4. Clearly, an addition of CO_2_ leads to substantial formation of similar surface carbonates which, to a large part, decompose again after CO_2_ is turned off.

**Fig. 5:**
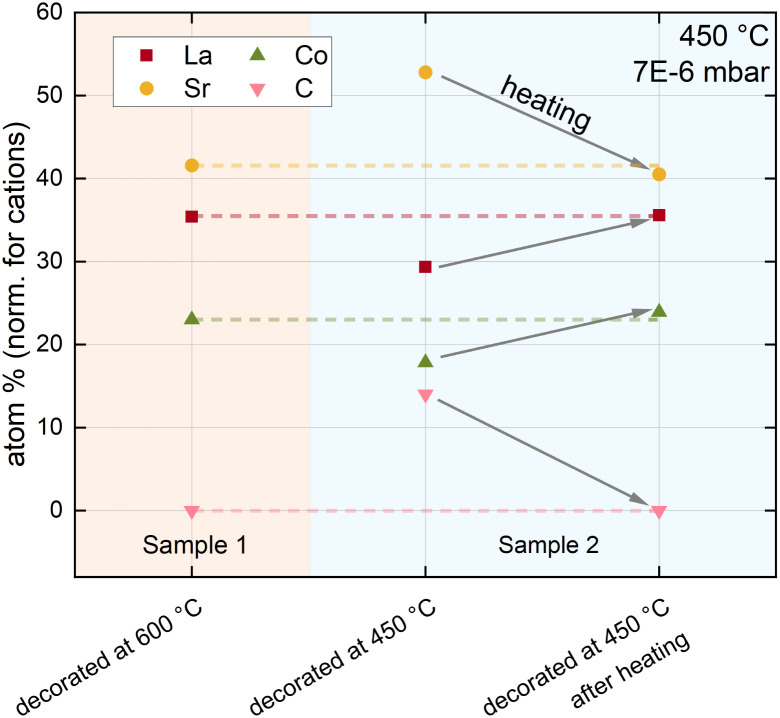
Surface chemistry of LSC decorated with 1 monolayer of SrO at high temperature compared with LSC decorated with 1 monolayer SrO at low temperature before and after heating to 600 °C inside the XPS chamber. All measurements were performed at 450 °C and 7 × 10^−6^ mbar oxygen.

These results are further supported by a comparison between LSC decorated at high temperatures and LSC decorated at low temperatures (see Fig. 5). A carbonate covered LSC (decorated at low temperatures) was first heated to 600 °C inside of the XPS chamber and subsequently measured at 450 °C. After this procedure, the surface chemistry drastically changed and, even more importantly, now completely resembled the surface chemistry of LSC decorated at high temperatures. Hence, the results are in excellent agreement with i-PLD results, where LSC decorated at low temperatures adopts the surface exchange kinetics of LSC decorated at high temperatures after heating to 600 °C.

### Non-ambient XRD study of SrO

**Fig. 6:**
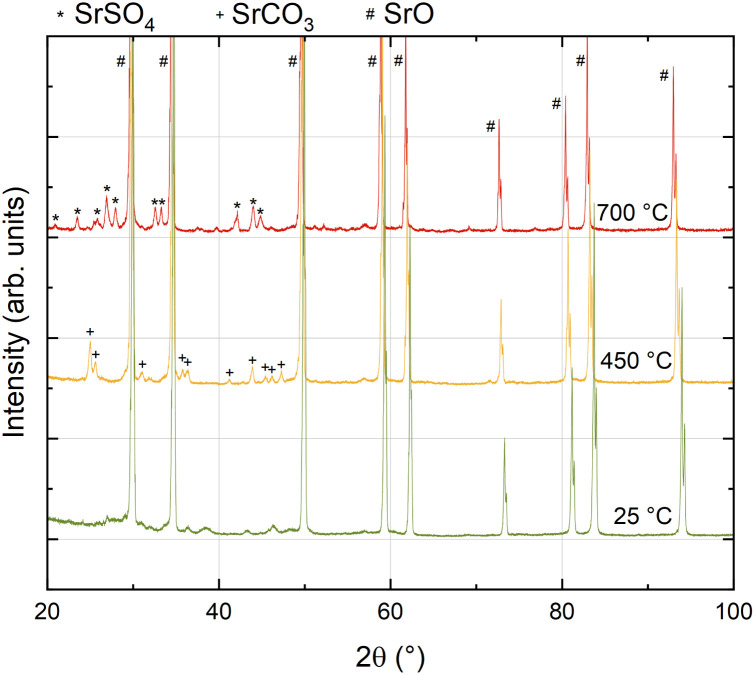
Non-ambient XRD measurements of SrO powder with 0.2 mbar O_2_ in N_2_ background gas.

As a complementary approach, non-ambient XRD measurements in controlled atmosphere and at elevated temperature were performed on the same SrO powder that was used for the PLD target (Fig. 6). The first diffractogram was recorded at room temperature immediately after calcining and exhibits almost exclusively SrO peaks, together with some very small peaks which could not be assigned conclusively to a specific secondary phase. Upon heating to 450 °C in 0.2 mbar O_2_ in N_2_ background gas, clear indications of a secondary phase formation appear in the diffractogram and all additional reflexes could be attributed to SrCO_3_, showing that even traces of CO_2_ in a nominally pure measurement atmosphere are sufficient to transform considerable amounts of SrO powder to SrCO_3_ at 450 °C. Upon further heating to 700 °C, the carbonates disappear and, simultaneously, a new phase appears which can be unambiguously identified as SrSO_4_.

This is in good agreement with recent results, confirming that measurement gases may also contain substantial amounts of sulphur impurities in the higher ppb range^39,40^ which readily adsorb on oxides and form surface sulphates, thus inducing a strong performance degradation on LSC surfaces. Also, this sulphate secondary phase is stable upon cooling. At this point it is important to emphasize that similar processes also take place in a standard *ex situ* impedance setup in measurement gas, though rather involving adsorbates than secondary phase formation (see below). Only the very clean atmosphere during our i-PLD measurements^40^ facilitated the observation of the electrochemical properties of real SrO decorations.

### Addition of CO_2_ during i-PLD measurements

**Fig. 7:**
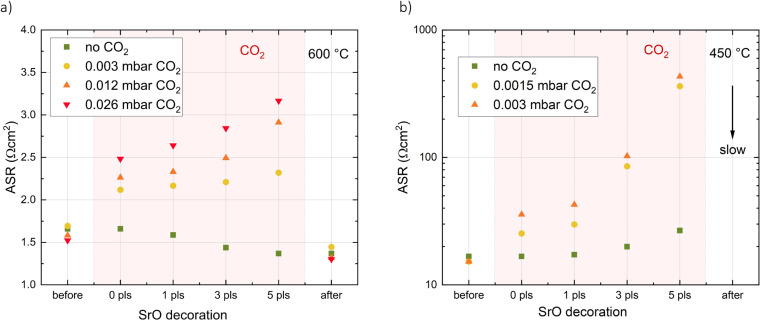
Effect of *in situ* decoration from a Sr-containing target on the area specific surface exchange resistance of a dense LSC thin film at (a) high (600 °C) and (b) low (450 °C) temperatures in 0.04 mbar O_2_ for different amounts of additional CO_2_ introduced into the chamber. The arrow indicates a slow but continuous recovery of the area specific surface exchange resistance after CO_2_ was turned off.

To complete the picture of the LSC surface chemistry during i-PLD, the CO_2_ addition performed in XPS experiments was repeated during i-PLD decoration experiments (Fig. 7). Again, the results obtained during i-PLD and XPS are in excellent agreement, with the addition of CO_2_ leading to a resistance increase of the oxygen surface exchange reaction (corresponding to the carbonate formation seen during XPS). When performed at 600 °C, the resistance increase is only minor and upon turning off the CO_2_ flow, the oxygen exchange kinetics improve rapidly, even beyond the initial point (indicating that the dominating surface species is again SrO). At lower temperatures, the resistance increase is much more pronounced as decomposition takes much longer and the resistance only decreases very slowly upon turning off the CO_2_ flow. Due to the continuous change of the surface exchange resistance at these conditions, the absolute resistance could not be evaluated and the process is indicated by a downward arrow in Fig. 7b.

From the combination of i-PLD, XPS and XRD measurements, we can clearly conclude that trace amounts of CO_2_ in measurement gases lead to carbonate formation on LSC at 450 °C which in turn causes an increased oxygen exchange resistance as is observed during i-PLD. At high temperatures, carbonates are not stable on LSC surfaces and leave behind SrO, which improves the catalytic activity of the surface and leads to a decrease of the surface exchange resistance (observable in i-PLD), even beyond the value without Sr-decoration.

## Discussion

In the previous section it was shown that Sr decoration of LSC can induce opposite effects on the oxygen exchange kinetics and therefore on the surface exchange resistance observed by impedance spectroscopy. In the following, we discuss possible reasons for this behavior and consider the implications of these results for the investigation of the oxygen exchange reaction on mixed electronic and ionic conductors.

### Beneficial and detrimental effects of surface Sr

The presented combination of measurements resolves the reasons behind the apparent contradictions in previous literature. Our i-PLD measurements showed that the effect of Sr decoration on the oxygen exchange kinetics strongly depends on temperature and atmosphere during decoration and measurement. The experiments of Rupp *et al.*, describing detrimental effects of Sr,^33^ were performed at relatively low temperatures and high oxygen partial pressures (decoration and impedance measurement). We thus strongly suspect that the resulting surface layer was not SrO, but rather carbonate-based. This would explain the observed decrease of the oxygen exchange kinetics, similar to the low-T decorations presented here. The decorations of Mutoro *et al.*^25^ were deposited and investigated at higher temperatures. Hence, most likely SrO species were present on the surface, at least partially explaining the accelerated oxygen exchange kinetics. This corresponds to the high-T decorations presented here, which also have a beneficial effect on the surface exchange resistance of LSC thin film electrodes. In addition, due to the large amount of decorated material, (La,Sr)CoO_3−*δ*_/(La,Sr)_2_CoO_4±*δ*_ interfaces, as suggested by Mutoro *et al.*, might also contribute to the substantially increased oxygen exchange coefficient.^25^ Please note that a positive effect of SrO also requires absence of SrSO_4_ formation due to sulphur impurities (we suspect SrSO_4_ formation as one of the main causes of degradation in thin film studies exploring Sr segregation^40^). These results not only clarify the effect of surface Sr on LSC, but are also in excellent agreement with the concept of surface acidity, with CO_2_ and SO_2_ (as acidic oxides) inhibiting the oxygen exchange on LSC surfaces and with SrO (as a basic oxide) increasing their performance (see below for an extended discussion of the correlation of oxygen exchange kinetics and surface acidity).

An important conclusion from the so far presented results is that Sr segregation *per se*, contrary to the widespread belief, initially does not have a detrimental impact on the oxygen exchange kinetics of LSC and related materials. However, Sr-rich surfaces in the presence of (even only trace amounts of) acidic species like SO_2_ or CO_2_ lead to the formation of sulphate or carbonate adsorbates and to a strong degradation of the oxygen exchange kinetics. It is also noteworthy that, based on our results, we strongly suspect these acidic adsorbates as the starting point for long-term degradation processes including particle formation and a substantial alteration of the host lattice.^40,41^ This theory is further substantiated by recent results, showing that it is possible to recover strongly degraded surfaces by heat-treatment and to dissolve segregated Sr from secondary phases (such as SrSO_4_ or SrCO_3_) back into the host material.^42^

### SrO *vs.* SrCO_3_ on LSC surfaces

While it is clear that carbonate species are present on low-T decorated LSC surfaces and upon intentional addition of CO_2_, a discussion of their correlation with the oxygen exchange kinetics requires further information about the detailed surface chemistry. A simple approach to this problem from a thermodynamic perspective might suggest that the surface of Sr-decorated LSC is covered with a full secondary phase of either SrO or SrCO_3_, depending on the environmental conditions during deposition and measurement. Different surface phases may then explain the different oxygen exchange kinetics at the two decoration temperatures. The two phases can be transformed into each other *via*:

1 SrO + CO_2_(g) ⇌ SrCO_3_,

and this was also investigated in our XRD measurements. The reaction itself is exergonic, *e.g.* with Δ*G*^0^ = −92 kJ mol^−1^ at 600 °C.^43^ According to bulk thermodynamic considerations, for given environmental conditions, a certain threshold p(CO_2_) for carbonate formation exists. This threshold p(CO_2_) is around 7 × 10^−6^ bar at 600 °C and 5 × 10^−7^ bar at 450 °C. As the oxygen partial pressure during i-PLD itself amounts to 4 × 10^−5^ bar (implying that the p(CO_2_) is several orders of magnitude below that), this strongly suggests, that no SrCO_3_ bulk formation should take place in our i-PLD studies, neither at 450 °C, nor at 600 °C. Accordingly, our decoration induced phenomena exceed the simple picture of secondary bulk phase formation. Please note that such a thermodynamic picture is indeed valid for the non-ambient XRD measurements of SrO powder, where due to the long measurement times and high gas pressures actual bulk secondary phases of SrCO_3_ and SrSO_4_ are formed. This also holds for long-term degradation processes of LSC surfaces in ambient pressure atmospheres where particle formation processes are observed.^44^

### Carbonate adsorbates and their effects on oxygen exchange kinetics

Instead of secondary phase formation, our hypothesis to explain the processes observed upon Sr decoration is based on CO_2_ adsorbates on the LSC surface. In agreement with literature, where CO_2_ adsorbates on similar materials have been frequently described,^13,29,31^ this hypothesis is able to explain many observed results:

– LSC decorated with Sr at low temperatures exhibits significantly higher signatures of carbon compared to pristine LSC. Upon heating to 600 °C, the carbon signal disappears completely and the surface chemistry fully resembles high temperature decorated LSC. This is caused by the desorption of carbonate-like CO_2_ adsorbates at higher temperatures.

– As was further seen in XPS measurements, intentional addition of CO_2_ to the gas feed yields a strong signature of carbonates in the signals of C and O, similar as for LSC decorated at low temperature. This signature quickly fades after removing the additional CO_2_ from the gas, again supporting that the observed signature stems from adsorbates. Similar results were also obtained during i-PLD measurements, where the addition of CO_2_ resulted in a temporary increase of the oxygen exchange resistance.

– The amount of adsorbed CO_2_ as well as the desorption kinetics depend on the temperature and the amount of CO_2_ in the atmosphere (in the present case governed by the oxygen partial pressure or by intentionally added CO_2_). This explains the difference in the absolute increase of the surface exchange resistance and in the recovery kinetics for different amounts of added CO_2_ at different temperatures as well as the gradual transitions observed during temperature cycling of decorated LSC. Similarly, the amount of adsorbed CO_2_ increases when the total pressure during i-PLD is increased (Fig. 2c), accounting for the stronger improvement upon high temperature decoration at lower pressures.

**Fig. 8:**
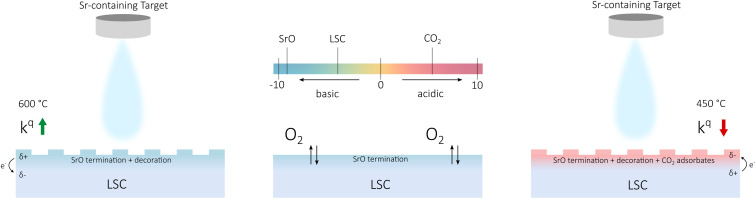
Schematic of the different situations encountered upon Sr decoration. Charge redistribution is indicated according to the relative acidity of the oxide termination. The effect on the oxygen surface exchange coefficient is indicated for the two cases. The surface acidity of LSC is calculated by proportionally adding up acidity values of its constituents.^27^

While a site blocking image as main effect of carbonate adsorption, as was proposed in literature,^13,29^ may explain observations at very high coverages, we suspect that it is not the complete picture. For lower coverages and for surface decorations in general, we suggest that also charging processes come into play. In literature, it is discussed that surface decorations alter the surface work function and induce a space charge zone with charge accumulation or depletion depending on the specific charge carrier.^26^ In addition, the induced surface potential may directly affect charge transfer steps of the oxygen exchange reaction and it might also influence reaction energetics such as adsorption equilibria.^27^ This is schematically shown in Fig. 8: for Sr decoration at high temperatures, a positive surface charge forms and the oxygen exchange kinetics are accelerated. For Sr decoration at low temperatures, negative charge accumulates on the CO_2_ adsorbates and the oxygen exchange kinetics are reduced. The exact mechanism how such charge redistribution processes affect the oxygen exchange reaction is yet unknown.

At this point, we want to emphasize the agreement of our results and hypothesis with the concept of surface acidity introduced by Nicollet *et al.*^26,27^ While CO_2_ adsorbates are acidic surface species and have a detrimental effect on the oxygen exchange kinetics, SrO (the result of Sr decoration at high temperatures) is a strongly basic oxide and improves oxygen surface exchange rates (see Fig. 8). Recently, we have also observed a similar phenomenon for sulphate groups adsorbed on LSC surfaces^40^ and we strongly suspect that we deal with similar effects in this study. For complementary evidence, LSC was decorated intentionally with an acidic oxide (SnO_2_), which also resulted in a significant increase of the surface exchange resistance (see Fig. SI 3†).

A remaining question is the influence of the amount of decorated material. On the one hand, substantially detrimental CO_2_ adsorption seems to require Sr decoration at low temperature. On the other hand, it is not straightforward why Sr decoration at high temperatures leads to a significant improvement of the surface exchange kinetics on an already SrO terminated LSC surface. In this context, we suspect that the decoration amplifies the properties of the termination, *i.e.* causes a higher similarity to real SrO (this is further supported by measurements where LSC was decorated with higher amounts of Sr at high temperature, which show that the optimal amount is reached at 1.5 monolayers after which the resistance starts to increase again, see Fig. SI 4†). Thereby, the surface basicity is increased, which makes the surface very prone to CO_2_ adsorption at low temperatures and which enhances the beneficial effect of a basic surface on the oxygen exchange kinetics at high temperatures. For a more in-depth investigation of these phenomena on an atomic scale, further analysis of the surface potential step and computational investigations of surface modifications are necessary, which go beyond the scope of this study.

## Conclusions

By the means of *in situ* impedance spectroscopy during pulsed laser deposition (i-PLD), we investigated the impact of Sr decoration of La_0.6_Sr_0.4_CoO_3−*δ*_ surfaces on their oxygen exchange kinetics. We discovered that decoration by deposition from a Sr containing target affects the oxygen exchange rate differently depending on the environmental conditions, *i.e.* temperature and oxygen partial pressure. At high temperatures (600 °C), Sr decoration improves the oxygen exchange kinetics, while at low temperatures (450 °C), it has the opposite effect and strongly deteriorates the oxygen exchange rate. Moreover, low-T decorated LSC fully recovers when heated to 600 °C and again shows the same properties as high-T decorated LSC. Near-ambient pressure XPS measurements revealed that low-T decorated LSC exhibits substantial amounts of surface carbonates which desorb when increasing the temperature until they fully disappear at 600 °C. These experiments resolve apparent contradictions in literature on the correlation of surface Sr and surface oxygen exchange kinetics. SrO surface decorations are beneficial for the oxygen exchange while SrCO_3_ surface species, formed at lower temperatures from SrO decoration and CO_2_ adsorbates from impurities in the measurement gas, have a detrimental effect. From a mechanistic perspective, these phenomena can be correlated with the surface acidity of the decorated surface and might affect various aspects of the oxygen exchange reaction, ranging from pure site availability over alteration of surface defect concentrations to the modification of the surface potential by charge redistribution. As a consequence, these results suggest that Sr segregation does not inherently inhibit the oxygen exchange reaction but requires acidic compounds like CO_2_ or SO_2_ to lead to commonly observed degradation phenomena.

## Conflicts of interest

There are no conflicts to declare.

## Supplementary Material

TA-011-D2TA09362F-s001

## References

[cit1] Adler S. B. (2004). Factors governing oxygen reduction in solid oxide fuel cell cathodes. Chem. Rev..

[cit2] Bucher E., Gspan C., Sitte W. (2015). Degradation and regeneration of the SOFC cathode material La_0.6_Sr_0.4_CoO_3−*δ*_ in SO_2_-containing atmospheres. Solid State Ionics.

[cit3] Fergus J. W. (2007). Materials challenges for solid-oxide fuel cells. Jom.

[cit4] Maguire E., Gharbage B., Marques F., Labrincha J. (2000). Cathode materials for intermediate temperature SOFCs. Solid State Ionics.

[cit5] Bouwmeester H. J., Kruidhof H., Burggraaf A. (1994). Importance of the surface exchange kinetics as rate limiting step in oxygen permeation through mixed-conducting oxides. Solid State Ionics.

[cit6] Januschewsky J., Ahrens M., Opitz A., Kubel F., Fleig J. (2009). Optimized La_0.6_Sr_0.4_CoO_3−*δ*_ Thin-Film Electrodes with Extremely Fast Oxygen-Reduction Kinetics. Adv. Funct. Mater..

[cit7] Siebenhofer M., Huber T. M., Friedbacher G., Artner W., Fleig J., Kubicek M. (2020). Oxygen exchange kinetics and nonstoichiometry of pristine La_0.6_Sr_0.4_CoO_3−*δ*_ thin films unaltered by degradation. J. Mater. Chem. A.

[cit8] Rupp G. M., Schmid A., Nenning A., Fleig J. (2016). The superior properties of La_0.6_Ba_0.4_CoO_3−*δ*_ thin film electrodes for oxygen exchange in comparison to La_0.6_Sr_0.4_CoO_3−*δ*_. J. Electrochem. Soc..

[cit9] Blum L., De Haart L. B., Malzbender J., Menzler N. H., Remmel J., Steinberger-Wilckens R. (2013). Recent results in Jülich solid oxide fuel cell technology development. J. Power Sources.

[cit10] Karageorgakis N. I., Heel A., Bieberle-Hütter A., Rupp J. L., Graule T., Gauckler L. J. (2010). Flame spray deposition of La_0.6_Sr_0.4_CoO_3−*δ*_ thin films: Microstructural characterization, electrochemical performance and degradation. J. Power Sources.

[cit11] Schrödl N., Bucher E., Egger A., Kreiml P., Teichert C., Höschen T., Sitte W. (2015). Long-term stability of the ITSOFC cathode materials La_0.6_Sr_0.4_CoO_3−*δ*_ and La_2_NiO_4+*δ*_ against combined chromium and silicon poisoning. Solid State Ionics.

[cit12] Bucher E., Yang M., Sitte W. (2012). *In situ* investigations of the chromium-induced degradation of the oxygen surface exchange kinetics of IT-SOFC cathode materials La_0.6_Sr_0.4_CoO_3−*δ*_ and La_0.58_Sr_0.4_Co_0.2_Fe_0.8_O_3−*δ*_. J. Electrochem. Soc..

[cit13] Zhao Z., Liu L., Zhang X., Wu W., Tu B., Ou D., Cheng M. (2013). A comparison on effects of CO_2_ on La_0.8_Sr_0.2_MnO_3+*δ*_ and La_0.6_Sr_0.4_CoO_3−*δ*_ cathodes. J. Power Sources.

[cit14] Koo B., Kim K., Kim J. K., Kwon H., Han J. W., Jung W. (2018). Sr segregation in perovskite oxides: why it happens and how it exists. Joule.

[cit15] Kwon H., Lee W., Han J. W. (2016). Suppressing cation segregation on lanthanum-based perovskite oxides to enhance the stability of solid oxide fuel cell cathodes. RSC advances.

[cit16] Kubicek M., Limbeck A., Frömling T., Hutter H., Fleig J. (2011). Relationship between cation segregation and the electrochemical oxygen reduction kinetics of La_0.6_Sr_0.4_CoO_3−*δ*_ thin film electrodes. J. Electrochem. Soc..

[cit17] Opitz A. K., Rameshan C., Kubicek M., Rupp G. M., Nenning A., Götsch T., Blume R., Hävecker M., Knop- Gericke A., Rupprechter G. (2018). *et al.*, The Chemical Evolution of the La_0.6_Sr_0.4_CoO_3−*δ*_ Surface Under SOFC Operating Conditions and Its Implications for Electrochemical Oxygen Exchange Activity. Top. Catal..

[cit18] Rupp G. M., Téllez H., Druce J., Limbeck A., Ishihara T., Kilner J., Fleig J. (2015). Surface chemistry of La_0.6_Sr_0.4_CoO_3−*δ*_ thin films and its impact on the oxygen surface exchange resistance. J. Mater. Chem. A.

[cit19] Druce J., Ishihara T., Kilner J. (2014). Surface composition of perovskite-type materials studied by Low Energy Ion Scattering (LEIS). Solid State Ionics.

[cit20] Chang K.-C., Ingram B., Ilavsky J., Lee S., Fuoss P., You H. (2017). Synchrotron X-ray studies of model SOFC cathodes, part I: thin film cathodes. Solid State Ionics.

[cit21] Niania M., Podor R., Britton T. B., Li C., Cooper S. J., Svetkov N., Skinner S., Kilner J. (2018). *In situ* study of strontium segregation in La_0.6_Sr_0.4_Co_0.2_Fe_0.8_O_3−*δ*_ in ambient atmospheres using high-temperature environmental scanning electron microscopy. J. Mater. Chem. A.

[cit22] Orikasa Y., Crumlin E. J., Sako S., Amezawa K., Uruga T., Biegalski M. D., Christen H. M., Uchimoto Y., Shao-Horn Y. (2014). Surface strontium segregation of solid oxide fuel cell cathodes proved by *in situ* depth-resolved X-ray absorption spectroscopy. ECS Electrochem. Lett..

[cit23] Anthony S. Y., Küngas R., Vohs J. M., Gorte R. J. (2013). Modification of SOFC cathodes by atomic layer deposition. J. Electrochem. Soc..

[cit24] Crumlin E. J., Mutoro E., Liu Z., Grass M. E., Biegalski M. D., Lee Y.-L., Morgan D., Christen H. M., Bluhm H., Shao-Horn Y. (2012). Surface strontium enrichment on highly active perovskites for oxygen electrocatalysis in solid oxide fuel cells. Energy Environ. Sci..

[cit25] Mutoro E., Crumlin E. J., Biegalski M. D., Christen H. M., Shao-Horn Y. (2011). Enhanced oxygen reduction activity on surface-decorated perovskite thin films for solid oxide fuel cells. Energy Environ. Sci..

[cit26] Nicollet C., Toparli C., Harrington G. F., Defferriere T., Yildiz B., Tuller H. L. (2020). Acidity of surface-infiltrated binary oxides as a sensitive descriptor of oxygen exchange kinetics in mixed conducting oxides. Nat. Catal..

[cit27] Nicollet C., Tuller H. L. (2022). Perspective on the Relationship between the Acidity of Perovskite Oxides and Their Oxygen Surface Exchange Kinetics. Chem. Mater..

[cit28] Hayd J., Ivers-Tiffée E. (2013). Detailed electrochemical study on nanoscaled La_0.6_Sr_0.4_CoO_3−*δ*_ SOFC thin-film cathodes in dry, humid and CO_2_-containing atmospheres. J. Electrochem. Soc..

[cit29] Yan A., Cheng M., Dong Y., Yang W., Maragou V., Song S., Tsiakaras P. (2006). Investigation of a Ba_0.5_Sr_0.5_Co_0.8_Fe_0.2_O_3−*δ*_ based cathode IT-SOFC: I. The effect of CO_2_ on the cell performance. Appl. Catal., B.

[cit30] Zhu Y., Sunarso J., Zhou W., Shao Z. (2015). Probing CO_2_ reaction mechanisms and effects on the SrNb_0.1_Co_0.9−*x*_Fe_*x*_O_3−*δ*_ cathodes for solid oxide fuel cells. Appl. Catal., B.

[cit31] Heel A., Holtappels P., Graule T. (2010). On the synthesis and performance of flame-made nanoscale La_0.6_Sr_0.4_CoO_3−*δ*_ and its influence on the application as an intermediate temperature solid oxide fuel cell cathode. J. Power Sources.

[cit32] Bucher E., Egger A., Caraman G., Sitte W. (2008). Stability of the SOFC cathode material (Ba,Sr)(Co,Fe)O_3−*δ*_ in CO_2_-containing atmospheres. J. Electrochem. Soc..

[cit33] Rupp G. M., Opitz A. K., Nenning A., Limbeck A., Fleig J. (2017). Real-time impedance monitoring of oxygen reduction during surface modification of thin film cathodes. Nat. Mater..

[cit34] Rupp G. M., Kubicek M., Opitz A. K., Fleig J. (2018). *In Situ* Impedance Analysis of Oxygen Exchange on Growing La_0.6_Sr_0.4_CoO_3−*δ*_ Thin Films. ACS Appl. Energy Mater..

[cit35] Siebenhofer M., Huber T., Artner W., Fleig J., Kubicek M. (2021). Substrate stoichiometry changes during pulsed laser deposition: a case study on SrTiO_3_. Acta Mater..

[cit36] Opitz A. K., Fleig J. (2010). Investigation of O_2_ reduction on Pt/YSZ by means of thin film microelectrodes: the geometry dependence of the electrode impedance. Solid State Ionics.

[cit37] Rameshan R., Nenning A., Raschhofer J., Lindenthal L., Ruh T., Summerer H., Opitz A. K., Martin Huber T., Rameshan C. (2020). Novel sample-stage for combined near ambient pressure x-ray photoelectron spectroscopy, catalytic characterization and electrochemical impedance spectroscopy. Crystals.

[cit38] Siebenhofer M., Riedl C., Schmid A., Limbeck A., Opitz A. K., Fleig J., Kubicek M. (2022). Investigating oxygen reduction pathways on pristine SOFC cathode surfaces by *in situ* PLD impedance spectroscopy. J. Mater. Chem. A.

[cit39] Schmid A., Nenning A., Opitz A., Kubicek M., Fleig J. (2020). High Oxygen Exchange Activity of Pristine La_0.6_Sr_0.4_FeO_3−*δ*_ Films and Its Degradation. J. Electrochem. Soc..

[cit40] Riedl C., Siebenhofer M., Nenning A., Schmid A., Weiss M., Rameshan C., Limbeck A., Kubicek M., Opitz A. K., Fleig J. (2022). *In situ* techniques reveal the true capabilities of SOFC cathode materials and their sudden degradation due to omnipresent sulfur trace impurities. J. Mater. Chem. A.

[cit41] Siebenhofer M., Haselmann U., Nenning A., Friedbacher G., Bumberger A. E., Wurster S., Artner W., Hutter H., Zhang Z., Fleig J. (2022). *et al.*, Surface Chemistry and Degradation Processes of Dense La_0.6_Sr_0.4_CoO_3−*δ*_ Thin Film Electrodes. J. Electrochem. Soc..

[cit42] Tripkovic D., Wang J., Küngas R., Mogensen M. B., Yildiz B., Hendriksen P. V. (2022). Thermally Controlled Activation and Passivation of Surface Chemistry and Oxygen-Exchange Kinetics on a Perovskite Oxide. Chem. Mater..

[cit43] Roine A. , HSC Chemistry 6 for Windows, Outokumpu Technology, 2006

[cit44] Ostrovskiy E., Huang Y.-L., Wachsman E. D. (2021). Effects of surface chemical potentials on cation segregation. J. Mater. Chem. A.

