# Supplementary material for: Improving and degrading the oxygen exchange kinetics of La0.6Sr0.4CoO3−δ by Sr decoration
Source: J Mater Chem A Mater. 2023 Feb 13;11(24):12827–36. doi: 10.1039/d2ta09362f (PMC10281333; doi:10.1039/d2ta09362f)
Supplement: TA-011-D2TA09362F-s001 [file TA-011-D2TA09362F-s001.pdf]

Electronic Supplementary Material (ESI) for Journal of Materials Chemistry A.
This journal is © The Royal Society of Chemistry 2023

Improving and degrading the oxygen exchange kinetics of $La_{0.6}Sr_{0.4}CoO_{3-\delta}$ by Sr decoration

Matthäus Siebenhofer*, Christoph Riedl*, Andreas Nenning, Werner Artner, Christoph Rameshan, Alexander Karl Opitz, Jürgen Fleig, Markus Kubicek

S.I 1. Surface morphology

AFM images were recorded of LSC surfaces (deposited at 600 °C in 0.04 mbar O_2) in the following three states: i) pristine, after deposition; ii) decorated with 1 monolayer of SrO at 600 °C; iii) decorated with 1 monolayer of SrO at 450 °C. The measurements show a granular structure for all thin films and no significant differences occurring upon decoration. We therefore conclude that the decorations do not agglomerate in particles but yield a homogenous surface coverage.

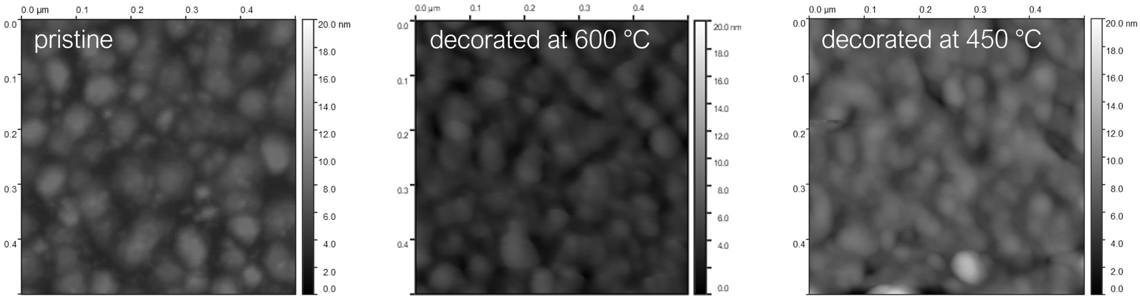

Figure 1: AFM images of a) pristine LSC, b) LSC decorated with one monolayer of SrO at 600 °C and c) LSC decorated with 1 monolayer of SrO at 450 °C.

S.I 2. XPS spectra of LSC decorated at high and low temperature

Fig. 2 shows the first XPS spectra at 600 °C in the Sr and in the O regions of LSC decorated with 1 monolayer of SrO at low temperature (450 °C) and high temperature (600 °C). While the Sr signal does not exhibit significant changes (as was expected, since the same amount of Sr was decorated on nominally identical thin films), the oxygen satellite feature corresponding to carbonates is still more pronounced for low temperature decorated LSC, indicating the not yet desorbed carbonates.

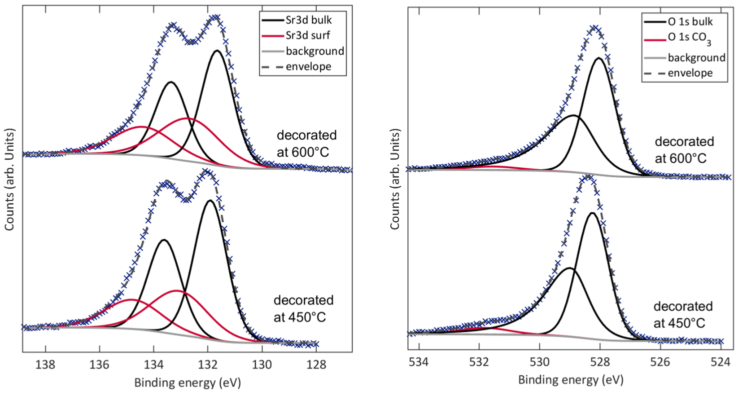

Figure 2: XPS spectra in the Sr and O regions of LSC decorated with SrO at high and low temperatures. It is clearly visible that the oxygen satellite feature corresponding to carbonate is more pronounced in the low temperature decorated case.

S.I 3. Decoration of LSC with an acidic oxide

To support the theory that SrO decoration at low temperatures leads to an acidic surface due to CO_2 adsorption, LSC was also decorated intentionally with an acidic oxide, SnO_2 (see Fig. 3). After 5 pulses, the surface exchange resistance had increased considerably. Please note, that the absolute deposited amount was not the same for 5 pulses SrO and SnO_2 (SnO_2 usually exhibits higher deposition rates), however, even after one pulse (<0.5 monolayers) the SnO_2 decoration had a detrimental effect on the oxygen exchange kinetics.

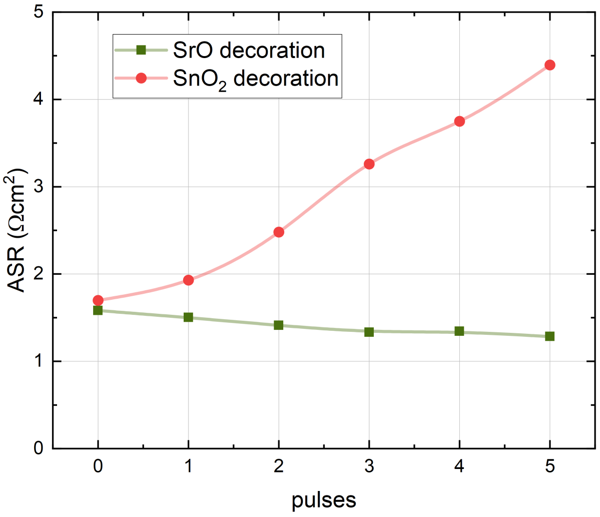

Figure 3: i-PLD decoration of LSC with SrO and SnO_2, one basic and one acidic oxide. Lines are a guide to the eye.

S.I 4. Saturation of decoration effects

LSC was decorated with higher amounts of SrO at high and low temperatures to see if the decoration effects saturates after a certain amount. For SrO decoration at high temperatures, a saturation is observed at around 1.5 monolayers, after which the area specific surface exchange resistance starts to increase again. For SrO decoration at low temperatures, the resistance increase is much more pronounced, similarly to the results of Rupp et al. [1] (see Fig. 4).

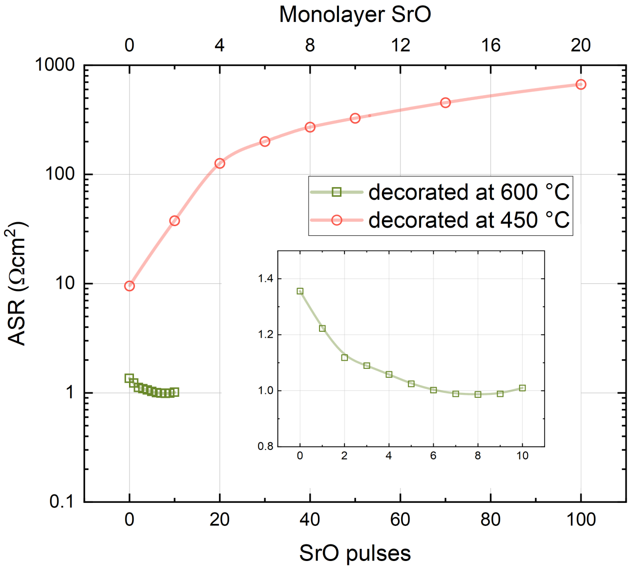

Figure 4: i-PLD decoration of LSC with larger amounts of SrO at 600 °C and 450 °C. The evolution of the area specific surface exchange resistance of high temperature decorated LSC is shown in the inset.

References

1. Rupp, G. M., Opitz, A. K., Nenning, A., Limbeck, A. & Fleig, J. Real-time impedance monitoring of oxygen reduction during surface modification of thin film cathodes. *Nature materials* **16,** 640–645 (2017).
